# Structural Insights into AQP2 Targeting to Multivesicular Bodies

**DOI:** 10.3390/ijms20215351

**Published:** 2019-10-28

**Authors:** Jennifer Virginia Roche, Veronika Nesverova, Caroline Olsson, Peter MT Deen, Susanna Törnroth-Horsefield

**Affiliations:** 1 Department of Biochemistry and Structural Biology, Lund University, PO Box 124, 221 00, Lund, Sweden; jennifer.roche@biochemistry.lu.se (J.V.R.); veronika.nesverova@biochemistry.lu.se (V.N.); caroline.olsson@biochemistry.lu.se (C.O.); 2 Department of Physiology, Radboud University Medical Centre, Geert Grooteplein-Zuid 10, 6525 GA Nijmegen, The Netherlands; peterdeen11@gmail.com

**Keywords:** aquaporin 2, water channel, multivesicular bodies, lysosomal trafficking regulator-interacting protein 5, trafficking, protein sorting

## Abstract

Vasopressin-dependent trafficking of AQP2 in the renal collecting duct is crucial for the regulation of water homeostasis. This process involves the targeting of AQP2 to the apical membrane during dehydration as well as its removal when hydration levels have been restored. The latter involves AQP2 endocytosis and sorting into multivesicular bodies (MVB), from where it may be recycled, degraded in lysosomes, or released into urine via exosomes. The lysosomal trafficking regulator-interacting protein 5 (LIP5) plays a crucial role in this by coordinating the actions of the endosomal sorting complex required for transport III (ESCRT-III) and vacuolar protein sorting 4 (Vps4) ATPase, resulting in the insertion of AQP2 into MVB inner vesicles. While the interaction between LIP5 and the ESCRT-III complex and Vps4 is well characterized, very little is known about how LIP5 interacts with AQP2 or any other membrane protein cargo. Here, we use a combination of fluorescence spectroscopy and computer modeling to provide a structural model of how LIP5 interacts with human AQP2. We demonstrate that, the AQP2 tetramer binds up to two LIP5 molecules and that the interaction is similar to that seen in the complex between LIP5 and the ESCRT-III component, charged multivesicular body protein 1B (CHMP1B). These studies give the very first structural insights into how LIP5 enables membrane protein insertion into MVB inner vesicles and significantly increase our understanding of the AQP2 trafficking mechanism.

## 1. Introduction

Most hormonally controlled cells respond to cellular and environmental signals by altering the abundance of specific membrane proteins in the plasma membrane through protein trafficking from storage vesicles. One of the best characterized examples of hormone-induced membrane protein trafficking is the vasopressin-dependent translocation of the water channel aquaporin 2 (AQP2) from storage vesicles to the apical membrane of the collecting duct principal cells. This process, which is governed by phosphorylation of the AQP2 C-terminus, results in increased apical membrane water permeability and is crucial for our ability to concentrate urine during periods of dehydration [1,2,3]. However, equally important for urine volume regulation is lowering the apical membrane abundance of AQP2 when hydration levels have been restored and its presence is no longer required. This is achieved through endocytosis, induced by short-chain ubiquitination of Lys 270 of the C-terminus, after which AQP2 is sorted within the endosomal trafficking system and stored again in vesicles, degraded in lysosomes, or released in urine as exosomes [4].

**Figure 1:**
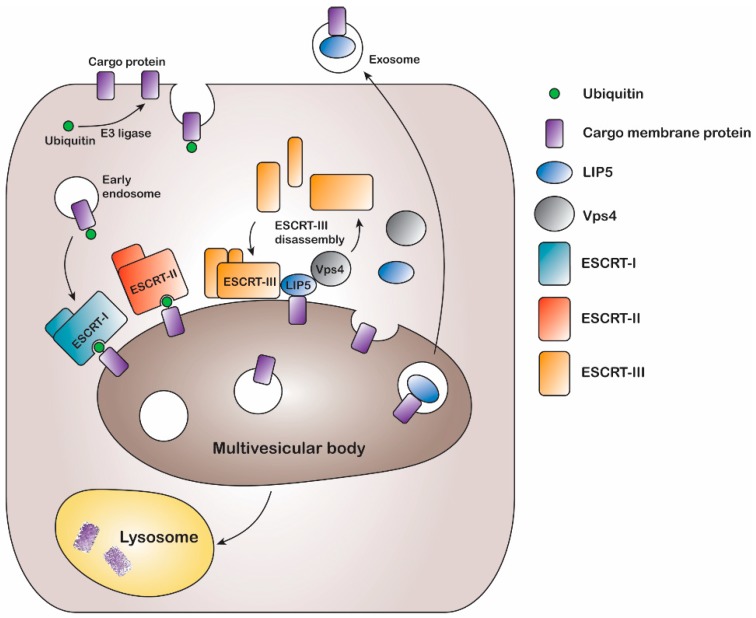
Schematic figure of the multivesicular body (MVB) membrane protein sorting mechanism. Cargo membrane proteins are ubiquitinated by E3 ubiquitin ligases and internalized through endocytosis. After sorting into early endosomes, the cargo membrane proteins interact with ESCRT-complexes I and II in the MVB limiting membrane and are inserted into MVB inner vesicles via actions of ESCRT-III and Vps4. The latter is coordinated by LIP5 which directly binds ESCRT-III and Vps4 and co-localizes with the membrane protein cargo inside the MVB inner vesicles. Finally, the MVBs may fuse with lysosomes, resulting in cargo protein degradation or they may fuse with the plasma membrane upon which the MVB inner vesicles are released extracellularly as exosomes. Black arrows indicate translocation events, attachment of ubiquitin and assembly/disassembly of the ESCRT-III complex.

The multivesicular body (MVB) sorting mechanism is a key component of the endocytic pathway and endosomal trafficking system. Following ubiquitination, endocytosed plasma membrane proteins are put in early endosomes from where they may be recycled back to the plasma membrane or sorted into late endosomal MVBs for degradation (Figure 1). This is often determined by removal or extended presence of the ubiquitin tag [5]. During MVB sorting, ubiquitinated plasma membrane proteins are targeted to intraluminal vesicles, which leads to degradation when the MVBs fuse with lysosomes or vacuoles [6,7]. Ubiquitination, a covalent modification of the membrane protein thus acts as a key signal for protein degradation; however, it is yet to be identified the exact stage at which this post-translational modification occurs [8,9,10].

**Figure 2:**
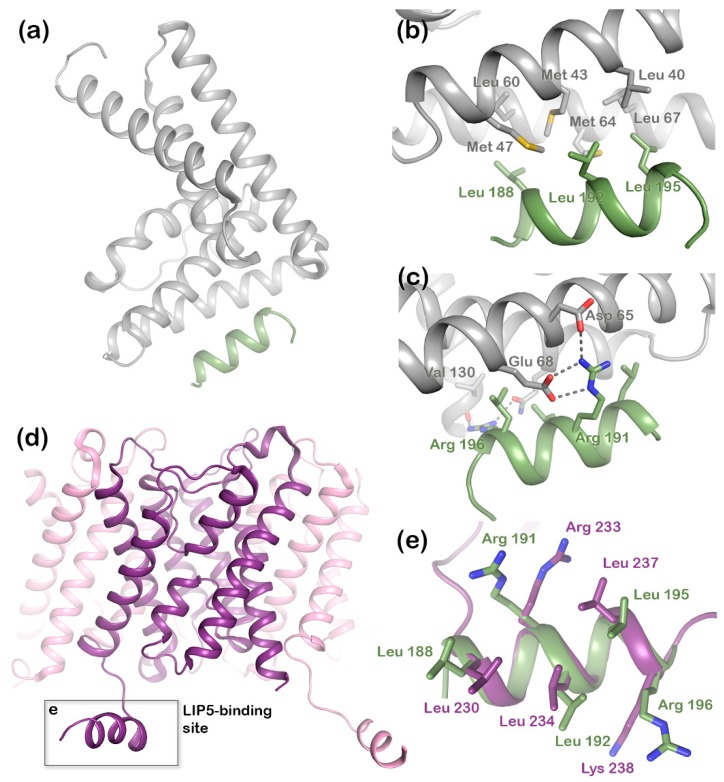
Structures of human lysosomal trafficking regulator-interacting protein 5 (LIP5) and aquaporin 2 (AQP2). (**a**) Crystal structure of the N-terminal domain of human LIP5 (grey, PDB code 4TXQ) in complex with a peptide from the ESCRT-III component, CHMP1B (green). Zoomed-in view of the LIP5-CHMP1B complex interface showing (**b**) hydrophobic and (**c**) hydrophilic interactions. (**d**) Crystal structure of human AQP2 tetramer (PDB code 4NEF) showing the classic AQP monomer fold (dark purple) with six transmembrane helices. The C-terminus extends into the cytoplasm and forms a short helix that is conserved in mammalian AQPs. (**e**) Overlay of the MIT-interacting motifs present in AQP2 C-terminal helix (purple) and the LIP5-binding segment of CHMP1B (green).

In eukaryotes, the MVB sorting mechanism is conserved and several factors mediating the reaction have been identified in both yeast and mammals. A series of class E vacuolar sorting proteins, endosomal sorting complexes required for transport (ESCRT) I-III, and associated proteins like vacuolar protein sorting (Vps) ATPases form the main part of the sorting machinery [7]. The ESCRT proteins play an important role in recognizing and sorting ubiquitinated membrane proteins via multiple endosomal trafficking pathways [5,6]. During MVB sorting, the ubiquitinated membrane protein cargo is inserted into intraluminal vesicles by sequentially binding ESCRT I, II, and III. Finally binding of the Vps4 ATPase results in membrane fission, ESCRT-III disassembly, and recycling of ESCRT machinery [11,12] (Figure 1). The MVB sorting machinery includes the lysosomal trafficking regulator-interacting protein 5 (LIP5), which directly binds to both the ESCRT-III complex and the Vps4 ATPase, thereby coordinating their actions [13]. LIP5 has been shown to interact with the charged multivesicular body protein (CHMP) components CHMP1B, CHMP2A, CHMP3 and CHMP5 of the ESCRT-III complex, via its N-terminal domain, whereas the binding site for the Vps4 ATPase is located in the C-terminal VSL (Vps4, SBP1, LIP5) domain [14,15,16,17,18,19]. The N-terminal domain of LIP5 (Figure 2a) has two microtubule interacting and trafficking (MIT) domains with a leucine collar that forms a hydrophobic core at the site of interaction [20]. Studies have demonstrated the canonical type-1 interaction between the MIT domain of LIP5 and an MIT-interacting motif (MIM1) in CHMP1B (Figure 2b,c) and a second high-affinity interaction between the second LIP5 MIT domain and an MIM element of CHMP5. Although the binding of LIP5 to the MIM1-containing ESCRT-III proteins and Vps4 is independent in vitro, the necessity for stable Vps4 complexes suggests that the interactions are coupled in vivo [13]. Furthermore, it has been shown that in mammals both the N- and C-terminal domains of LIP5 are required for Vps4 stimulation [20].

In addition to interacting with ESCRT-III and Vps4, LIP5 has also been shown to directly bind the membrane protein cargo. This includes AQP2, which has been shown to co-localize with LIP5 in MVB inner vesicles and for which the interaction with LIP5 facilitates its lysosomal degradation [12]. AQP2 interacts with LIP5 via the proximal part of the C-terminus [12,21], a region that forms a short cytoplasmic alpha helix that is conserved in mammalian AQP structures (Figure 2d). In the crystal structure of human AQP2, this C-terminal helix displays an unusual flexibility, occupying different positions in each monomer of the AQP2 homotetramer (Figure S1) [22]. In contrast, its position across the cytoplasmic protein interface is conserved within the tetramer in all other mammalian AQP structures [23,24,25,26]. The AQP2 C-terminal helix has a strong amphipathic character and contains a leucine-rich MIM1 consensus motif, perfectly matching the LIP5 interaction site found in the ESCRT-III component, CHMP1B (Figure 2e) [20]. We recently showed that AQP2 directly binds LIP5 in a phosphorylation-dependent manner and that the interaction is allosterically controlled by AQP2 phosphorylation at sites distal to the proposed LIP5-binding site [21].

Here we further investigate the interaction between AQP2 and LIP5 and present a structural model for the AQP2-LIP5 complex. We demonstrate that LIP5 binds AQP2 via its N-terminal domain as previously proposed [21], with up to two LIP5 molecules binding per AQP2 tetramer. Assuming that the interaction is mediated via the AQP2 MIM1 motif in the C-terminal helix, we performed a computer docking experiment using the HADDOCK2.2 docking server [27]. The docking resulted in two high-scoring models, one of which perfectly fits the binding mode seen in the LIP5-CHMP1B complex. The effect of point mutations within the proposed binding sites confirmed this model as the one best representing the AQP2-LIP5 complex. Our results give the first structural insights into how LIP5 interacts with cargo membrane proteins during MVB sorting, thereby deepening our understanding of the molecular details behind one of the key processes in regulating plasma membrane protein abundance in general and that of AQP2 trafficking in particular.

## 2. Results

### 2.1. Human LIP5 Interacts with AQP2 via Its N-Terminal Domain

The ability of full-length LIP5 to bind AQP2 has been demonstrated using several methods, including yeast two-hybrid assays [12], microscale thermophoresis [21,28], and fluorescence anisotropy [28]. Due to the presence of a MIM1-motif in the AQP2 C-terminus, it is believed that the interaction is mediated by the MIT modules in the LIP5 N-terminal domain [21]. To verify this, we used fluorescence spectroscopy to study the interaction using the full-length human LIP5 (FL-LIP5) and a truncated construct containing only the N-terminal domain of LIP5 (ND-LIP5, residues 1–162). For this purpose, human AQP2 was fluorescently labeled with Alexa 488. Since LIP5 binding has been shown to be dependent on site-specific AQP2 phosphorylation and AQP2 can be phosphorylated in the overproduction host (*Pichia pastoris*) [21], dephosphorylation was carried out prior to labeling.

**Figure 3:**
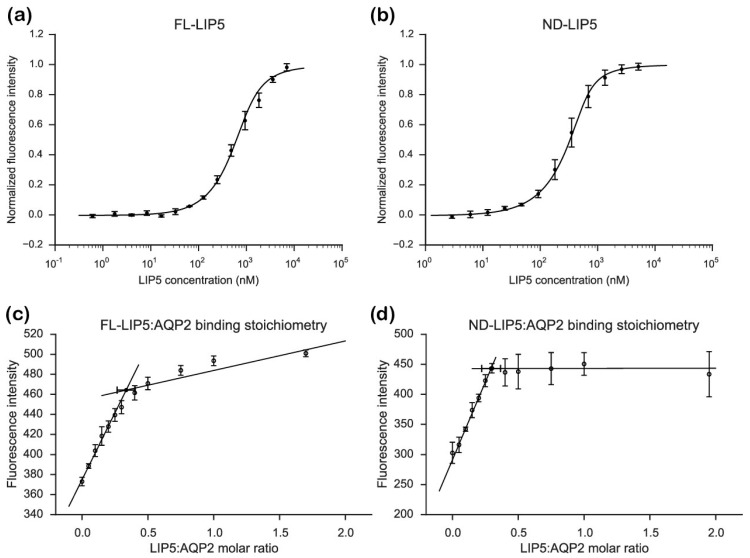
Fluorescence spectroscopy analysis of the interaction between LIP5 and AQP2. Binding curves for (**a**) full-length LIP5 (FL-LIP5) and (**b**) the N-terminal domain of LIP5 (ND-LIP5). Stoichiometry plot for (**c**) FL-LIP5 and (**d**) ND-LIP5 binding to AQP2. The molar ratio, determined from the saturation point (closed circle), was determined to be 0.42 ± 0.08 and 0.29 ± 0.07 for FL-LIP5 and ND-LIP5, respectively. Each curve was obtained from three independent titration series for which each point was measured three times.

Labeled AQP2 was titrated with unlabeled FL-LIP5 or ND-LIP5 and the fluorescence was monitored. For both the constructs, an increase in fluorescence could be observed with increasing concentrations of LIP5 (until saturation was reached), resulting in binding curves from which the dissociation constant K_d_ could be determined (Figure 3). This is similar to what has been shown in previous binding experiments between AQP2 and LIP5 using the same dye [28] and suggests that the fluorescence signal from Alexa 488 is dequenched upon LIP5 binding. As seen in Figure 3b, ND-LIP5 bound AQP2 with somewhat higher affinity than full-length LIP5 (Figure 3a), the K_d_ was determined to be 64 ± 34 nM and 237 ± 66 nM, respectively, a difference that was shown to be statistically significant. The K_d_ for full-length LIP5 is comparable to what we have observed previously by both fluorescence anisotropy (320 ± 100 nM) and microscale thermophoresis (191 ± 43.2 nM) [21,28].

### 2.2. The AQP2 Tetramer Binds up to Two LIP5 Molecules

To investigate how many LIP5 molecules bind to the AQP2 tetramer, we determined the stoichiometry of the complex. In the stoichiometry experiment, increasing amounts of unlabeled FL-LIP5 or ND-LIP5 were added to the cuvette containing labeled AQP2 at a concentration well above the K_d_ value for the interaction. This resulted in a saturation curve, where the characteristic kink could be observed once a saturating concentration of LIP5 was obtained. The stoichiometry could be determined by calculating the exact position of this kink along the *x*-axis by fitting the data to two linear equations, thereby giving the concentration of LIP5 necessary to saturate the complex.

The saturation point was calculated to be at 0.42 ± 0.08 molar ratio for FL-LIP5:AQP2 (Figure 3c) and 0.29 ± 0.07 for ND-LIP5:AQP2 (Figure 3d). Statistical analysis did not show any significant difference between these two ratios. This means that, on average, one to two molecules of LIP5 bind to one AQP2 tetramer.

### 2.3. Computer Modeling of the LIP5-AQP2 Complex

The mode in which LIP5 binds the MIM1 motifs has been structurally characterized in the crystal structure of the LIP5 N-terminal domain bound to a peptide from the ESCRT-III component, CHMP1B [20]. In this complex, Leu188, Leu192, and Leu195 in CHMP1B make hydrophobic interactions with hydrophobic residues from both helices in the first MIT domain of LIP5 (Figure 2b). In addition, polar interactions can be seen between Arg191 in CHMP1B and Asp65 and Glu68 in LIP5. The three leucine residues and the arginine in CHMP1B are part of a MIM1 consensus motif (D/EXXLXXRLXXLR/K) that is also present in the C-terminal helix of AQP2 (Figure 2d). We therefore postulated that the C-terminal helix of AQP2 would interact with the N-terminal domain of LIP5 in a similar manner.

To evaluate this, we performed a computer docking experiment where the crystal structures of AQP2 (PDB code 4NEF) and the LIP5 N-terminal domain (PDB code 4TXQ) were uploaded to the HADDOCK2.2 (high ambiguity driven protein-protein docking) server [27]. The LIP5 residues involved in the interaction with CHMP1B and the MIM1 motif in the AQP2 C-terminal helix were used as input to drive the docking. In the crystal structure of AQP2, where each monomer has a different C-terminal conformation, the C-terminal helix of monomer C makes hydrophobic interactions with symmetry-related molecules in the crystal packing [22] (Figure S1). We reasoned that this supports its ability to participate in protein-protein interactions and therefore used this monomer in the docking experiment.

**Figure 4:**
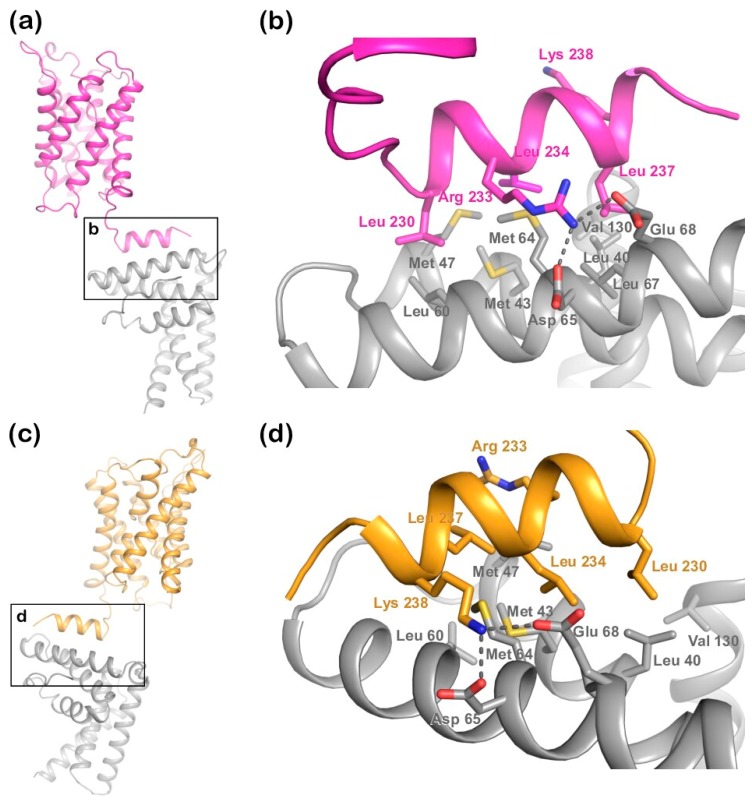
Structural representation of the two best docking models from HADDOCK. (**a**) Cluster 3 solution with the C-terminal helix of AQP2 (magenta) binding to the first MIT-domain of the LIP5 N-terminal domain (grey); (**b**) zoomed-in view of the interacting surface in (**a**) showing that in this solution, LIP5 binds AQP2 in a similar manner as in the LIP5-CHMP1B complex (Figure 2b,c); (**c**) cluster 2 solution showing how the AQP2 (orange) C-terminal helix binds the LIP5 N-terminal domain in the opposite orientation; (**d**) Zoom-in view of the interacting surface in (**c**). In (**b**,**d**), all residues corresponding to the binding interface in the LIP5-CHMP1B complex are shown in stick representation. Predicted hydrogen bonds are depicted as dotted lines.

The docking resulted in 164 structures, which grouped into 11 clusters. The top 10 clusters were evaluated based on their HADDOCK score (Table S1) as well as the similarity between the top solution in the cluster and the binding mode observed in the LIP5-CHMP1B complex (Figure 2 and Figure S2). Based on this, cluster 2 (highest HADDOCK score) and cluster 3 (third highest HADDOCK score) were judged as the most likely docking models (Table 1). In the two clusters, LIP5 binds the AQP2 C-terminal helix in a similar manner, but in opposite directions (Figure 4). As in the LIP5-CHMP1B structure (Figure 2b,c), both models show Leu230, Leu234, and Leu237 of the AQP2 MIM1 motif fitting into a hydrophobic pocket formed by Met43, Met47, Met64, and Leu67 in the first LIP5 MIT-domain (Figure 4b,d). In cluster 3, hydrogen bonds are formed between Arg233 in the AQP2 MIM1 motif and Asp65 and Glu68 of LIP5, thus reproducing the hydrogen bonds seen between the corresponding residues in the LIP5-CHMP1B complex (Figure 2c). An additional hydrogen bond is also suggested to form between the main chain carboxyl group of AQP2 Leu240, which lies outside the MIM1 consensus motif, and Lys71 in LIP5. In contrast, Arg233 does not participate in hydrogen bonds in cluster 2, instead the second basic residue in the MIM1 motif, Lys238, interacts with Asp65 and Glu68 in LIP5. Taken together, while cluster 2 is the better model in terms of HADDOCK score, cluster 3 is significantly more similar to the known LIP5-MIM1 binding mode.

### 2.4. Validation of Docking Model by Mutational Analysis

**Figure 5:**
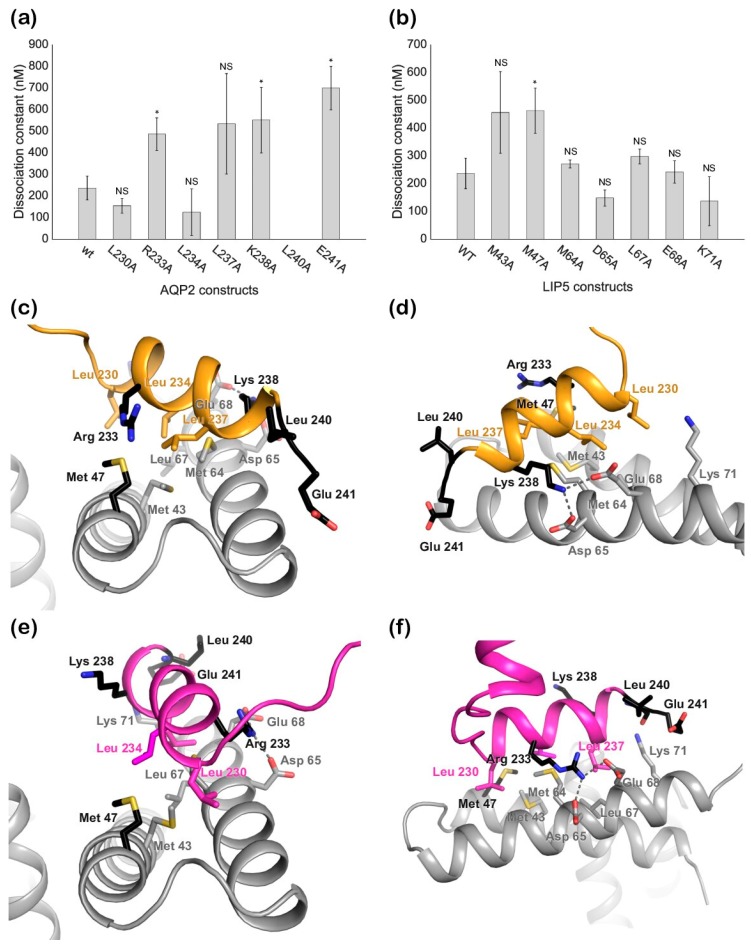
Mutational analysis of the best HADDOCK docking solutions. Bar charts showing the dissociation constants (K_d_) for the interaction between (**a**) LIP5 and AQP2 wild-type/mutants and (**b**) AQP2 and LIP5 wild-type/mutants. * indicates *p* < 0.05 (*n* = 3), NS indicates no significance; (**c**,**d**) structural representation of the interaction sites in cluster 2; (**e**,**f**) Structural representation of the interaction sites in cluster 3. Mutated residues which lower the affinity of the interaction or abolish it completely are shown in black. All residues that were probed by mutation are shown in stick representation. Predicted hydrogen bonds are depicted as dotted lines.

To validate which one of the two docking models are more likely to be correct, we made a set of binding-site single mutants of LIP5 and AQP2 that would allow us to discriminate between the two possible binding modes. For this purpose, Met43, Met47, Met64, Asp65, Leu67, Glu68, and Lys71 in full-length LIP5 and Leu230, Arg233, Leu234, Leu237, Lys238, Leu240, and Glu241 in AQP2 were individually mutated to alanine residues. The affinity of the mutants towards their wild-type interaction partner was determined using fluorescence spectroscopy as described above (Figures S3 and S4), the results of which are summarized in Table 2 and Figure 5a,b. Four mutations in AQP2 (R233A, K238A, L240A, and E241A) significantly reduced the affinity toward LIP5 as compared to wild-type AQP2 (Table 2 and Figure 5a). Of these, the L240A mutation completely abolished the interaction. In LIP5, the M47A mutant had lower affinity towards AQP2, whereas the affinities of all other mutants were not significantly different from wild-type LIP5 (Table 2 and Figure 5b).

Next, we analyzed the location of the mutation sites that reduced the affinity of the complex in the two docking solutions (Figure 5c–f). In cluster 2, only one of the AQP2 mutation sites (Lys238) is involved in the interaction with LIP5, while the other three (Arg233, Leu240, and Glu241) are pointing away from the interaction site (Figure 5c,d). In contrast, three out of four residues lie within the interacting surface in cluster 3; only Lys238 is located outside (Figure 5e,f). Likewise, Met47, the only residue in LIP5 for which mutation affected the interaction, is able to participate in hydrophobic interactions in cluster 3, but not in cluster 2. Based on these results, as well as the similarity with the binding mode observed in the LIP5-CHMP1B complex (Figure 2b,c), we therefore conclude that cluster 3 is the more accurate model.

## 3. Discussion

The ESCRT-III complex and Vps4 ATPase have been described as constituting a minimal membrane fission machine that is required for all known ESCRT-III dependent processes. LIP5 plays a central role in this by binding to both the components, coordinating the actions of the two and stimulating the ATPase activity of Vps4. Structural studies of N-terminal domain of LIP5 in complex with the ESCRT-III components CHMP1B and CHMP5 [20], as well the yeast LIP5 homolog Vta1 C-terminal domain in complex with Vps4 [29,30], have given detailed structural insights into how LIP5 controls the ESCRT-III/Vps4 membrane fission machinery. In contrast, very little is known about how LIP5 binds cargo proteins and the mechanism behind LIP5 and cargo protein co-localization inside MVB intraluminal vesicles is unknown. Our model of the LIP5-AQP2 complex gives the very first structural insight into this process.

Structural studies of the ternary complex of LIP5 and the ESCRT-III components CHMP1B and CHMP5 have shown that CHMP1B binds to LIP5 MIT1 domain (Figure 2), whereas CHMP5 binds to the MIT2 domain as well as the MIT1-MIT2 interface. While the interaction between LIP5 and CHMP1B does not have any effect on the Vps4 activity, binding of CHMP5 to LIP5 has been shown to inhibit its stimulatory effect on Vps4. It has therefore been proposed that the role of CHMP1B is to recruit LIP5 to the MVB limiting membrane. The interaction between LIP5 and CHMP5 is proposed to lead to conformational changes within LIP5 that release a suggested interaction between the LIP5 N-terminal domain and Vps4 that is necessary for Vps4 stimulation [20]. Here we show that AQP2 binds to the same LIP5 motif as CHMP1B and in a very similar manner. This suggests that cargo proteins, such as AQP2, may directly compete with CHMP1B for interacting with LIP5. If the interaction with a cargo leads to further conformational changes around the MIT1-MIT2 interface, binding of CHMP5 may also be affected, potentially fully releasing LIP5 from the ESCRT-III complex after which ESCRT-III disassembly could occur (Figure 1).

From our stoichiometry analysis we conclude that, on average, one AQP2 tetramer binds up to two LIP5 molecules (Figure 3c,d). Since full-length LIP5 is known to form a tight dimer through its C-terminal domain [13], we speculate that this corresponds to one LIP5 dimer binding up to two AQP2 C-terminal helices. As seen in our hypothetical structural model of full-length LIP5 (Figure S5), the N- and C-terminal domains of LIP5 are separated by a very long and flexible linker, and dimerization through the C-terminal domain would result in an elongated molecule with one N-terminal domain at either end. These two N-terminal domains could then interact with the C-terminal helix from two AQP2 monomers within the same tetramer. This is somewhat similar to how aquaporin 0 (AQP0) has been proposed to interact with calmodulin (CaM), in which case, two C-terminal helices of AQP0 binds one molecule of CaM, albeit within the same binding pocket [31]. Although our data for FL-LIP5 does not allow us to distinguish between the dimer binding one or two AQP2 molecules within the same tetramer, the similar stoichiometry obtained for ND-LIP5 supports two N-terminal domains binding the tetramer simultaneously. Alternatively, two LIP5 dimers binding to one AQP2 tetramer may interact with a second AQP2 tetramer through a sandwich effect. To discriminate between these scenarios, structural data for the AQP2-LIP5 complex will be needed.

Although the AQP2 tetramer contains four possible LIP5-binding sites, our data shows that a maximum of two of those are occupied at any given time. Since the crystal structure of the AQP2 tetramer shows high C-terminal structural variability (Figure S1) [22], it is possible that the conformation that allows the C-terminal helix to bind LIP5 is prohibited from existing in all four monomers at the same time. It is also possible that the elongated structure of LIP5, in particular in its dimeric form (Figure S5), limits the number of LIP5 molecules that are able to bind for steric reasons. Similarly, steric reasons could explain why the smaller ND-LIP5 binds AQP2 with higher affinity than full-length LIP5. Since the N-terminal domain does not exist on its own in the cell, the physiological significance of the difference in affinity is difficult to assess. One explanation could be that the LIP5-dimer is able to adopt different conformations, resulting in modulation of the affinity between LIP5 and AQP2. Such conformational differences may be related to the LIP5 C-terminal domain binding Vps4 and be part of coordinating the membrane fission event with membrane protein cargo recruitment. It must be pointed out, however, that this scenario is highly speculative and the difference in affinity between FL-LIP5 and ND-LIP5 could simply be an artefact from extracting a smaller domain from the full-length context.

Our mutational studies show that single mutations of hydrophobic residues within the AQP2 MIM1 motif (Leu230, Leu234, and Leu237) did not affect LIP5 binding, whereas mutations of hydrophilic residues did (Arg233 and Lys238). It seems reasonable that this is due to a single mutation of hydrophobic residues not being enough to disrupt binding, as long as a sufficiently large hydrophobic pocket is formed, whereas the hydrophilic interactions must be maintained. However, our studies also point at residues immediately outside the MIM1 motif (Leu240 and Glu241) as being important for the interaction. Further structural studies will be necessary to discern which of these residues participate directly in the interaction or if their mutation has knock-on effects that change the overall structure of the C-terminus.

To conclude, we present a structural model of the AQP2-LIP5 complex, giving the very first structural insight into how membrane proteins are recruited to MVB inner vesicles. This provides a platform for further studies that will significantly advance our understanding of the MVB sorting machinery as well as the mechanism for downregulating AQP2 apical membrane abundance during urine volume regulation. 

## 4. Materials and Methods 

### 4.1. LIP5 Cloning, Expression and Purification

DNA coding for the N-terminal domain of human LIP5 (residues 1–162) was amplified by PCR from pET3a encoding a previously described FL-LIP5-construct [28] and using 5′-GGGTTCCATATGGCGGCGCTGGCGCCGCTGCCG-3′ (sense) and 5′-CTTGGATCCTTAATGGT GATGATGATGGTGATGGTGCTGAAAATACAAGTTTTCGGTAGTTGGGATATCGTAATCCGGGGTTTCACCGTTTTTCAG-3′ (antisense) as forward and reverse primers, respectively. The NdeI and BamHI restriction digestion sites were used for cloning into the pET3a vector. The final construct contained a C-terminal hexa-histidine tag preceded by a tobacco etch virus (TEV) protease site. A spacer (DYDIPTT) was introduced between the last ND-LIP5 amino acid and the TEV site to ensure efficient proteolytic cleavage. The FL-LIP5 construct had been cloned previously [28] and contains the same tag and TEV-cleavage site and linker. LIP5 alanine mutants of the potential LIP5 binding sites (M43A, M47A, L60A, M64A, D65A, L67A, E68A, and K71A) were created using a megaprimer protocol [32]. 

All LIP5 constructs were expressed in BL21*(DE3) *Escherichia coli* cells as previously described [33]. The cells were grown in LB media containing 50 µg/mL ampicillin. For large-scale expression, 2 L of LB medium was inoculated from an overnight culture and the cells were grown at 37 °C with constant shaking at 170 rpm for 1.5–2 h. When the cells reached a mid-log phase (OD_600_ between 0.6–0.8), the temperature was reduced to 30 °C and protein expression was induced with 0.5 mM IPTG. The culture was grown for additional 3.5 to 4 h, after which the cells were harvested at 6000 g for 15 min. The bacterial pellet was collected and stored at −20 °C for further use.

The cells were thawed and resuspended in 50 mL lysis buffer (20 mM Tris pH 8, 250 mM NaCl, 10 mM imidazole, 5% glycerol). One cOmplete^TM^ protease inhibitor tablet (Roche, Switzerland) or 1 mM PMSF was added to the lysis buffer to inhibit protease activity. The cell suspension was subjected to sonication for ten minutes while placed on ice, with one-minute lysis followed by one-minute rest intervals. Cell debris was removed by centrifugation at 18,000× *g* for 30 min at 4 °C. The supernatant was carefully decanted and filtered using a 0.45 µm sterile filter before loading onto a 5 mL nickel HisTrap column (GE Healthcare, United States). Prior to loading, the column was equilibrated with the lysis buffer. The loosely bound impurities were removed by washing the column with a lysis buffer containing 30 mM or 100 mM imidazole. A lysis buffer supplemented with 250 mM imidazole was used to elute bound LIP5.

Samples from the HisTrap column were analyzed on SDS-PAGE and fractions corresponding to LIP5 were pooled and concentrated using a 10 kDa molecular weight cut-off Vivaspin concentration tube (GE Healthcare, United States) before loading onto a size exclusion chromatography (SEC) column. The SEC column, Superdex 200 10/300 (GE Healthcare, United States), was equilibrated with LIP5 SEC buffer (20 mM Tris pH8, 150 mM NaCl, 1 mM DTT) onto which approximately 500 µL of concentrated sample was loaded. The sample was eluted based on size and the fractions corresponding to LIP5 were analyzed on SDS-PAGE. The fractions corresponding to LIP5 were pooled and concentrated as mentioned above. The sample was directly used for experimental purposes or flash frozen with liquid nitrogen (5–10% glycerol added) and stored at −80 °C.

### 4.2. AQP2 Cloning, Expression, and Purification

The wild-type (WT) construct contains a full-length sequence of AQP2, preceded by an uncleavable His-tag on the N-terminus. AQP2 alanine mutants of the potential LIP5-binding sites (L230A, R233A, L234A, L237A, K238A, L240A, and E241A) were created using a megaprimer protocol [32].

The expression of human AQP2 wild-type and the mutants was done in *Pichia pastoris* as described previously [22]. Cells were grown in basal salt media in a 3L fermenter (Belach Bioteknik AB, Sweden). On depletion of glycerol, the cells were fed with additional 200–300 mL glycerol to increase biomass, after which the cells were slowly fed with methanol for 36–48 h for protein expression. The cells were harvested at 6000× *g* for 20 min at 4 °C resulting in 250–300 g of wet cells per litre of culture.

For membrane preparation, 50–100 g of cells was thawed and resuspended in a phosphate buffer (50 mM potassium phosphate pH 7.5, 5% glycerol, 2 mM EDTA) and lysed using a bead beater. Prior to bead beating, 1 mM PMSF was added. The cells were lysed for 30 s followed by a 30 s pause and this was repeated for a total of 12 cycles. The cell lysate was centrifuged at 16,000 g for 40 min at 4 °C to pellet down cell debris. The supernatant was further ultracentrifuged at 100,000 g for 1 h to isolate crude membranes. These membranes were homogenized and washed with a urea buffer (5 mM Tris-HCl pH 9.5, 4 M urea, 2 mM EDTA) and centrifuged for 2 h; followed by a membrane buffer wash (20 mM Tris-HCl pH 8, 20 mM NaCl, 10% glycerol, 2 mM EDTA, 1 mM PMSF) and centrifuged 1 h 15 min at 100,000× *g* at 4 °C. The washed membranes were finally resuspended in 20 mM Tris-HCl pH 8, 20 mM NaCl, 10% glycerol (0.5 g of membranes per mL of buffer), flash-frozen in liquid nitrogen, and stored at −80 °C until further use.

The thawed membranes were solubilized in a 1:1 ratio with a solubilization buffer (20 mM Tris pH 8, 300 mM NaCl, 10% glycerol, 4% octyl glucose neopentyl glycol (OGNG, Anatrace, United States). One cOmplete™ EDTA-free protease inhibitor cocktail tablet (Roche, Switzerland) or 1 mM PMSF was added to the solubilization mixture (50 mL). The solubilization buffer was added dropwise to the membrane suspension until the final OGNG concentration was 2%. The membranes were left to solubilize for one hour at 4 °C with continuous stirring. The non-solubilized material was spun down by centrifugation at 100,000× *g* for 45 min. The supernatant was decanted and supplemented with 10 mM imidazole before loading onto a HisTrap column which was equilibrated with buffer A (20 mM Tris pH 8, 300 mM NaCl, 0.2% OGNG). The column was washed with 5 column volumes of buffer A supplemented with 75 mM imidazole prior to elution with 300 mM imidazole. Fractions corresponding to the elution peak were pooled and concentrated using 30 kDa molecular weight cut-off Vivaspin concentration tubes (GE Healthcare, United States). The concentrated sample (about 500 µL) was passed through a 0.45 µm spin filter and loaded on an SEC column (Superdex 200 10/300, GE Healthcare, United States) equilibrated with buffer A. The fractions containing AQP2 were pooled and concentrated as above and kept on ice for immediate use or flash frozen with liquid nitrogen and stored at −80 °C for future use.

### 4.3. AQP2 Dephosphorylation and Labeling

All purified AQP2 samples were dephosphorylated using alkaline phosphatase for 2 h at 30 °C prior labeling as described previously [21]. This was done in order to remove the effect of phosphorylation on binding affinity in subsequent experiments.

The labeling of cysteine residues of AQP2 constructs with the cysteine-reactive dye C5 maleimide Alexa 488 (ThermoFisher Scientific, United States) was done according to the manufacturer’s instructions. A twenty-fold molar excess of the dye in 20 mM Tris-HCl pH 8, 300 mM NaCl, and 0.2% OGNG was added and the mixture was incubated overnight at 4 °C. The labeled protein was separated from free dye by desalting using a PD-10 or Superdex 200 10/300 column. The eluted fractions were analyzed based on absorbance at 280 nm (A_280_) using Nanodrop (the absorbance from the dye itself was accounted for) and the fractions with the highest A_280_ were used directly or concentrated and stored at −80 °C.

### 4.4. Fluorescence Spectroscopy

Fluorescence measurements were carried out in a LS-50B fluorometer (PerkinElmer, United States). The concentration of labeled AQP2 was kept around 1 μM (corresponding to 4 times the expected K_d_ for the wild-type complex) to ensure a high enough fluorescence signal. A 1:1 (wild-type) or 2:1 (mutants) dilution series of LIP5 in a buffer containing 20mM Tris-HCl pH 8, 300 mM NaCl, and 0.2% OGNG was prepared. For each sample in the dilution series, a fixed volume was sequentially added to the cuvette containing labeled AQP2. After each addition, the sample was left to equilibrate for 1 min before the measurements were taken. Five readings (LIP5 mutants) or three readings (AQP2 mutants) for each sample point were taken and the average total fluorescence was recorded. Each titration series was done in triplicates. The total fluorescence I_tot_ was calculated as:

(1) $$I_{tot}=\;I_{vv}\;+\;2I_{vh}$$

where I_vv_ is the intensity of vertically polarized light and I_vh_ corresponds to the intensity of horizontally polarized light [34].

For the stoichiometry measurements, a fixed volume of either full-length LIP5 or ND-LIP5 was sequentially added to a cuvette containing labeled AQP2. The concentration of labeled AQP2 was kept 20 times above the K_d_ of the interaction. The same buffers were used as above. The final LIP5 concentration in the cuvette corresponded to a molar LIP5:AQP2 ratio between 0 and 2. Each titration series was done in triplicate and each sample point was measured three times. The total fluorescence for each addition was determined as above.

### 4.5. Data Analysis

The fluorescence data was fitted with the following equations using the Python library, SciPy and the non-linear least squares method:

(2) $$y=S1+\left(S2-S1\right)\left(\frac{L_{Free}}{L_{Free}+K_{d}}\right)$$

(3) $$L_{Free}=0.5\;\left(L_{Tot}-P_{Tot}-K_{d}\right)+\sqrt{\frac{{\left(K_{d}+P_{Tot}-L_{Tot}\right)}^{2}}{4}+L_{Tot}K_{d\;}}$$

where S1 is the signal from the unbound state, S2 is the signal from the complex, L_Free_ and L_Tot_ are the free and total LIP5 concentrations, respectively, P_Tot_ is the total concentration of fluorescently labeled AQP2, and K_d_ is the dissociation constant.

Where fitting could be performed, each run of the triplicate was normalized to set the unbound state to 0 and bound state to 1. The mean of normalized triplicate data at each sample point was fitted again with the same equations. In the fit, each point was weighted by the deviation of the averaging. Standard deviation for each calculated K_d_ corresponds to the square root of variance within the final fit. The dilution of labeled AQP2 on subsequent addition of unlabeled LIP5 was accounted for during the fitting.

To evaluate if the K_d_ values obtained for ND-LIP5 as well as AQP2 and LIP5 mutants were significantly different from the K_d_ values for full-length wild-type proteins, a statistical two-sample T-test for means was employed. A *p*-value lower than 0.05 (indicated as an asterisk on the respective bar charts in Figure 5a,b) was used as the cut-off for statistical significance.

The averaged data from each stoichiometry measurement was fitted to two linear equations using a weighted fit, while keeping the total error as low as possible. The total error was calculated as the sum of the squares of all residuals. The x-coordinate for the intercept of the two lines was determined from the following equation:

(4) $$x=\frac{b2-b1}{m1-m2}$$

where m is the slope and b is the y-intercept in the general linear equation *y* = m*x* + b. The error propagation method was used to calculate the standard deviation for the intercept x-coordinate. As described above, a statistical two-sample T-test for means was performed to determine if the resulting stoichiometries for FL-LIP5 and ND-LIP5 were significantly different from each other.

### 4.6. Computer Docking

The docking of AQP2 to the N-terminal domain of LIP5 was performed in HADDOCK2.2 [27] using the crystal structures of human AQP2 (PDB code 4NEF, monomer C) and the N-terminal domain of human LIP5 (PDB code 4TXP). LIP5 residues involved in the interaction between LIP5 and CHMP1B (Tyr36, Leu67, Met64, Leu60, Met47, Leu40, Lys71, Glu68, Asp65, and Gln44) and AQP2 residues in the MIM motif (Leu230, Arg233, Leu234, Leu237, and Lys238) were used as active residues to drive the docking. The docking solutions were evaluated based on the HADDOCK score and Z-score (the number of standard deviations the HADDOCK score of a given cluster is separated from the mean of all clusters) as well as similarity with the known binding mode for LIP5-MIM1 interactions.

### 4.7. Modeling of Full-Length LIP5

To create the full-length model of LIP5, a homology model of the C-terminal VSL-domain was made in Swiss-MODEL [35] using the structure of Vta1 from *Saccharomyces cerevisiae* as the template (PDB code 5XMK). A model for the structurally uncharacterized linker domain connecting the N-terminal domain with the VSL domain was obtained from the structure prediction server, EVfold [33]. As expected, the linker domain, which is rich in glycine and proline residues, is predicted to not have any secondary structure elements (Figure S6). The crystal structure of the N-terminal domain (PDB code 4TXP), the predicted linker domain, and the VSL domain homology model were assembled in Coot [36] and the connecting bonds were regularized.

## Figures and Tables

**Table 1:** HADDOCK docking statistics for the two best clusters. All energies are given in kcal/mol. RMSD represents the root-mean-square deviation of the cluster from the overall lowest energy structure. The Z-score indicates how many standard deviations from the average the cluster is located in terms of score (the more negative, the better).

	Cluster 2	Cluster 3
HADDOCK score	−80.3 ± 3.7	−65.9 ± 5.1
Cluster size	26	22
RMSD (Å)	8.6 ± 0.3	6.2 ± 1.6
Van der Waals energy	−15.1 ± 3.1	−20.1 ± 6.1
Electrostatic energy	−381.1 ± 37.5	−269.1 ± 39.4
Desolvation energy	8.3 ± 6.6	5.6 ± 7.1
Restraints violation energy	26.7 ± 12.57	24.2 ± 21.40
Buried surface area (Å2)	1052.3 ± 52.0	992.8 ± 137.7
Z-score	−1.7	−0.6

**Table 2:** Binding affinities of the AQP2 and LIP5 constructs.

**LIP5 Construct**	**Binding Affinity (nM)**	**Error in Binding Affinity (nM)**
FL-LIP5	237.28	55.06
ND-LIP5	64.37	33.82
M43A	456.69	147.22
M47A	463.12	81.14
M64A	271.33	14.72
D65A	148.96	29.01
L67A	298.27	26.64
E68A	242.72	40.28
K71A	137.49	88.25
**AQP2 Construct**	**Binding Affinity (nM)**	**Error in Binding Affinity (nM)**
L230A	155.61	33.90
R233A	486.75	75.65
L234A	125.52	107.65
L237A	534.80	232.36
K238A	552.03	151.69
L240A	no binding	no binding
E241A	699.63	100.66

## Supplementary Materials

Supplementary Materials can be found at https://www.mdpi.com/1422-0067/20/21/5351/s1.

## Abbreviations

AQP	Aquaporin
CHMP	Charged multivesicular body protein
ESCRT	Endosomal sorting complex required for transport
FL	Full-length
IMAC	Immobilized metal affinity chromatography
LIP5	Lysosomal trafficking regulator-interacting protein 5
MIM	MIT-interacting motif
MIT-domain	Microtubule interacting and trafficking domain
MVB	Multivesicular body
ND	N-terminal domain
OGNG	Octyl glucose neopentyl glycol
PDB	Protein databank
SBP1	Suppressor of potassium transport growth defect-binding protein 1
VPS	Vacuolar protein sorting
VSL	Vps4, SBP1, LIP5
WT	Wild-type
