# Supplementary material for: Structural Insights into AQP2 Targeting to Multivesicular Bodies
Source: Int J Mol Sci. 2019 Oct 28;20(21):5351. doi: 10.3390/ijms20215351 (PMC6862464; doi:10.3390/ijms20215351)
Supplement: Supplementary file 1 [file ijms-20-05351-s001.zip › Supplementary files/Supplementary_IJMS_AQP2_MVB_190920.docx]

Supplementary Information

Structural insights into AQP2 targeting to multivesicular bodies

Jennifer Virginia Roche ^1†^, Veronika Nesverova ^1†^, Caroline Olsson ^1^, Peter MT Deen ^2^ and Susanna Törnroth-Horsefield ^1,^*

^1^ Department of Biochemistry and Structural Biology, Lund University, Sweden

^2^ Department of Physiology, Radboud University Medical Centre, Nijmegen, Netherlands

***** Correspondence: [susanna.horsefield@biochemistry.lu.se](mailto:susanna.horsefield@biochemistry.lu.se)

^†^ These authors contributed equally to this work


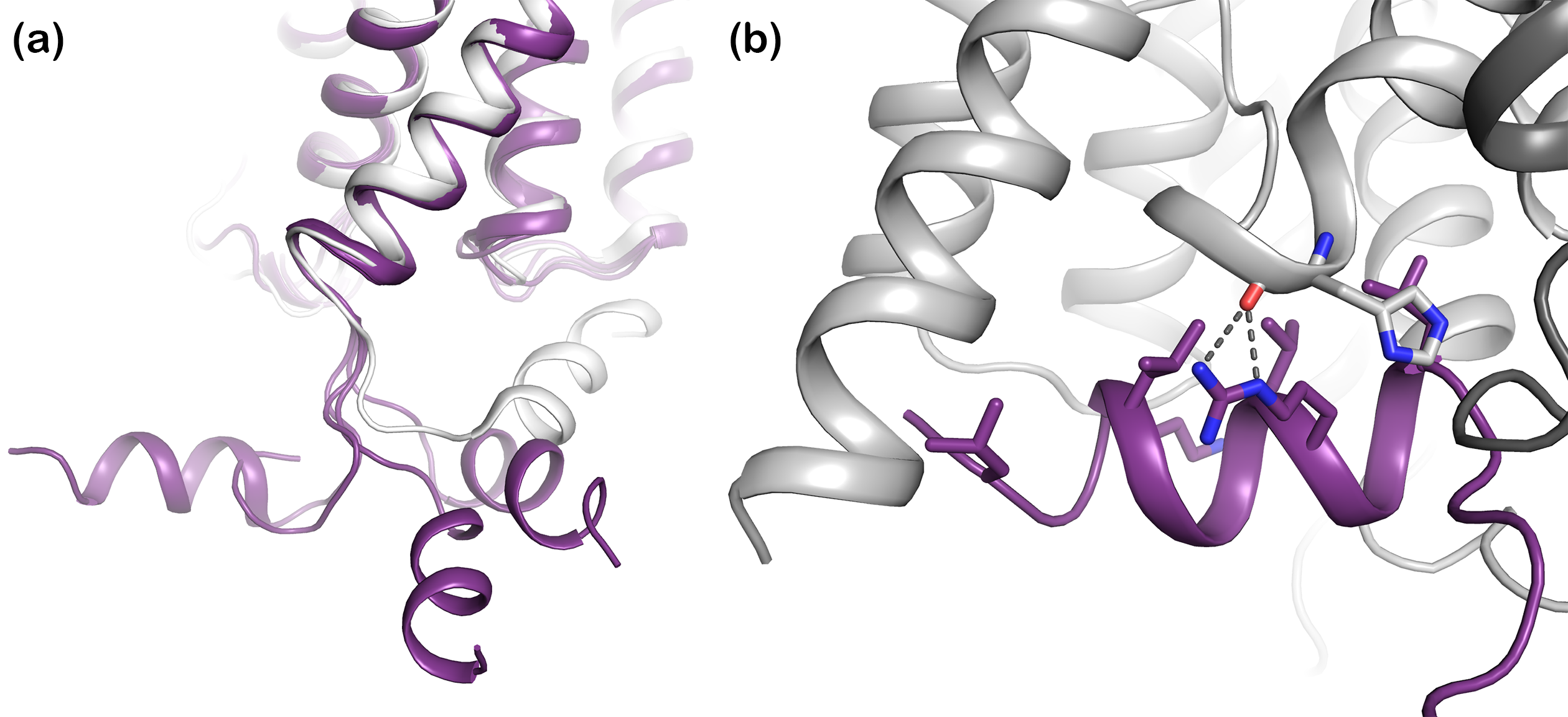


**Figure S1. Structural features of the AQP2 C-terminal helix. (a)** Overlay of the four different monomers in the crystal structure of AQP2 (purple, PDB code 4NEF) showing how the C-terminal helix adopts different conformations. The structure of human AQP5 (white, PDB code 3D9S) is also overlaid, representing the position of the C-terminal helix in the other mammalian AQP structures; **(b)** The MIM1 motif of the AQP2 C-terminal helix interacts with symmetry-related molecules in the human AQP2 crystal.


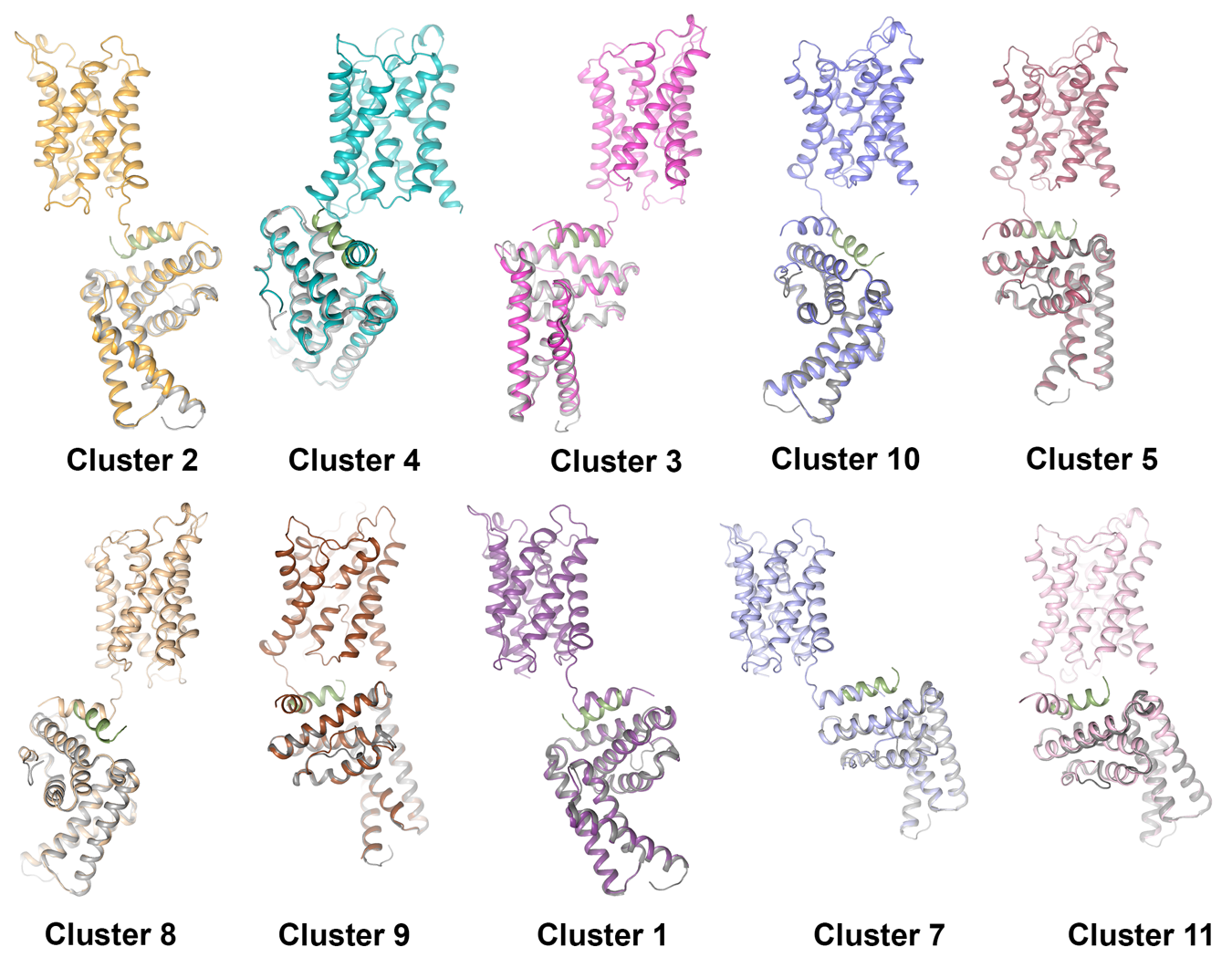


**Figure S2. Structural representation of all HADDOCK solution clusters.** For each cluster, the N-terminal domain of LIP5 has been overlaid on the LIP5-CHMP1B complex with the CHMP1B peptide containing the MIM1 motif shown in green.

**Figure S3. Binding curves for AQP2 mutants as obtained by fluorescence spectroscopy.** For L230A the first 3 points and the last point in the titration series were omitted in the fit. All measurements were done in triplicate.

**Figure S4. Binding curves for LIP5 mutants as obtained by fluorescence spectroscopy.** All measurements were done in triplicate.


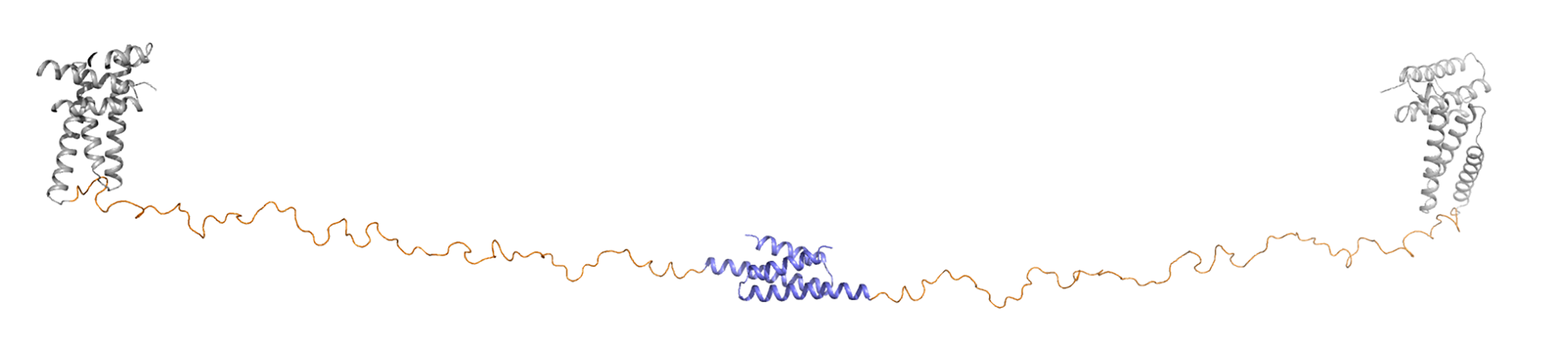


**Figure S5. Model of the full-length LIP5 dimer.** LIP5 dimerizes through its C-terminal domain (blue) while the N-terminal domain (grey) is available for other interactions. The two domains are separated by a long flexible linker (orange).

| **Table S1. HADDOCK docking statistics.** Detailed docking statistics for the top 10 clusters ordered from highest to lowest HADDOCK score. All energies are given in kcal/mol. RMSD represents the root mean square deviation of the cluster from the overall lowest-energy structure. The Z-score indicates how many standard deviations from the average the cluster is located in terms of score (the more negative the better). | | | | | | | | | |
| --- | --- | --- | --- | --- | --- | --- | --- | --- | --- |
| Cluster | HADDOCK score | Cluster size | RMSD (Å) | Van der Waals energy | Electrostatic energy | Desolvation energy | Restraints violation energy | Buried surface area (Å^2^) | Z-score |
| 2 | -80.3 +/- 3.7 | 26 | 8.6 +/- 0.3 | -15.1 +/- 3.1 | -381.1 +/- 37.5 | 8.3 +/- 6.6 | 26.7 +/- 12.57 | 1052.3 +/- 52.0 | -1.7 |
| 4 | -76.5 +/- 13.0 | 22 | 2.1 +/- 0.6 | -43.6 +/- 10.2 | -158.2 +/- 50.1 | -2.7 +/- 4.5 | 13.9 +/- 18.38 | 1362.5 +/- 157.8 | -1.4 |
| 3 | -65.9 +/- 5.1 | 22 | 6.2 +/- 1.6 | -20.1 +/- 6.1 | -269.1 +/- 39.4 | 5.6 +/- 7.1 | 24.2 +/- 21.40 | 992.8 +/- 137.7 | -0.6 |
| 10 | -59.1 +/- 7.3 | 5 | 9.5 +/- 0.6 | -18.1 +/- 1.1 | -283.5 +/- 17.4 | 15.4 +/- 5.5 | 4.3 +/- 2.32 | 810.3 +/- 52.7 | -0.1 |
| 5 | -57.3 +/- 2.8 | 18 | 9.5 +/- 0.4 | -18.8 +/- 3.1 | -305.3 +/- 38.0 | 19.7 +/- 2.0 | 27.9 +/- 23.79 | 878.3 +/- 44.5 | 0.0 |
| 8 | -53.1 +/- 8.4 | 7 | 7.7 +/- 0.7 | -20.6 +/- 3.4 | -256.6 +/- 59.0 | 16.6 +/- 7.0 | 22.8 +/- 15.82 | 903.1 +/- 59.0 | 0.3 |
| 9 | -52.1 +/- 14.4 | 6 | 9.8 +/- 0.7 | -19.5 +/- 3.2 | -253.1 +/- 100.1 | 15.2 +/- 7.6 | 28.0 +/- 36.91 | 963.1 +/- 113.7 | 0.4 |
| 1 | -52.0 +/- 5.8 | 35 | 10.6 +/- 0.9 | -15.4 +/- 5.1 | -259.0 +/- 35.0 | 11.8 +/- 5.1 | 33.8 +/- 16.58 | 885.4 +/- 50.3 | 0.4 |
| 7 | -41.0 +/- 2.0 | 8 | 13.0 +/- 0.5 | -22.6 +/- 3.0 | -156.0 +/- 21.4 | 10.7 +/- 3.9 | 21.5 +/- 16.68 | 976.0 +/- 55.8 | 1.2 |
| 11 | -35.8 +/- 11.2 | 5 | 8.5 +/- 0.5 | -14.4 +/- 2.7 | -195.7 +/- 29.6 | 12.7 +/- 4.6 | 50.7 +/- 41.20 | 793.4 +/- 63.7 | 1.6 |
