# Supplementary figures and images for: Structural Insights into AQP2 Targeting to Multivesicular Bodies
Source: Int J Mol Sci. 2019 Oct 28;20(21):5351. doi: 10.3390/ijms20215351 (PMC6862464; doi:10.3390/ijms20215351)

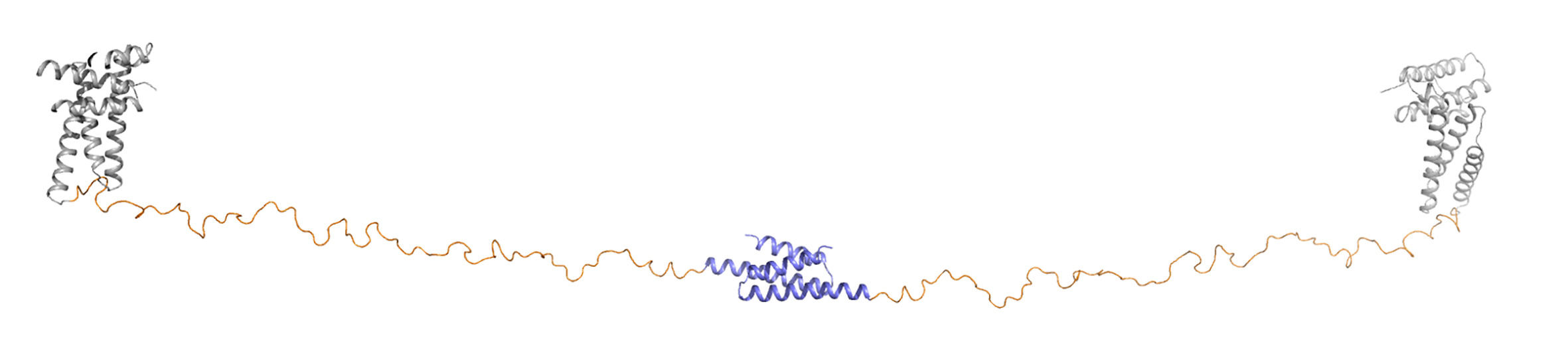

Supplement: Supplementary file 1 [file ijms-20-05351-s001.zip › Supplementary files/Fig S5.tif]

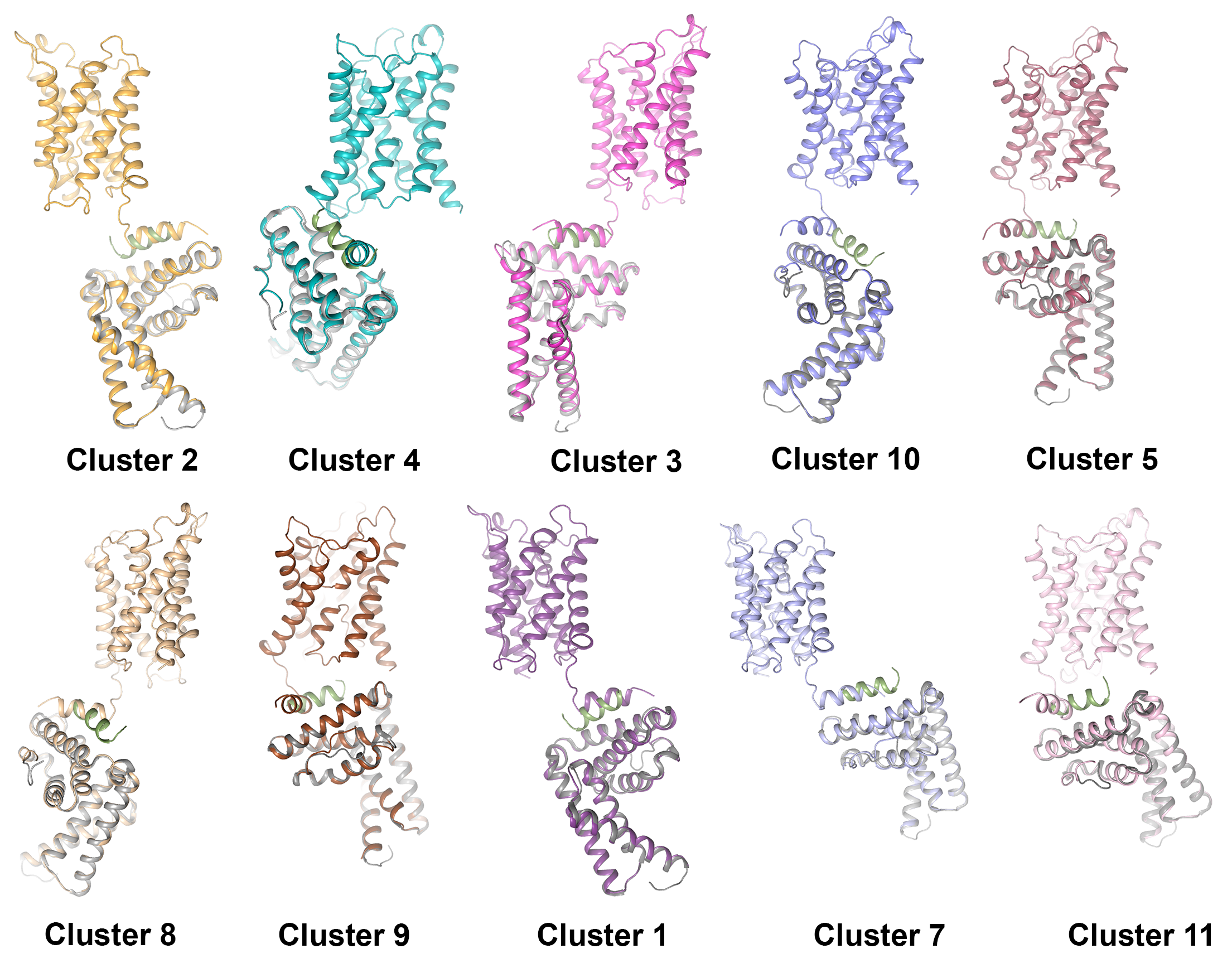

Supplement: Supplementary file 1 [file ijms-20-05351-s001.zip › Supplementary files/Figure S2.tif]

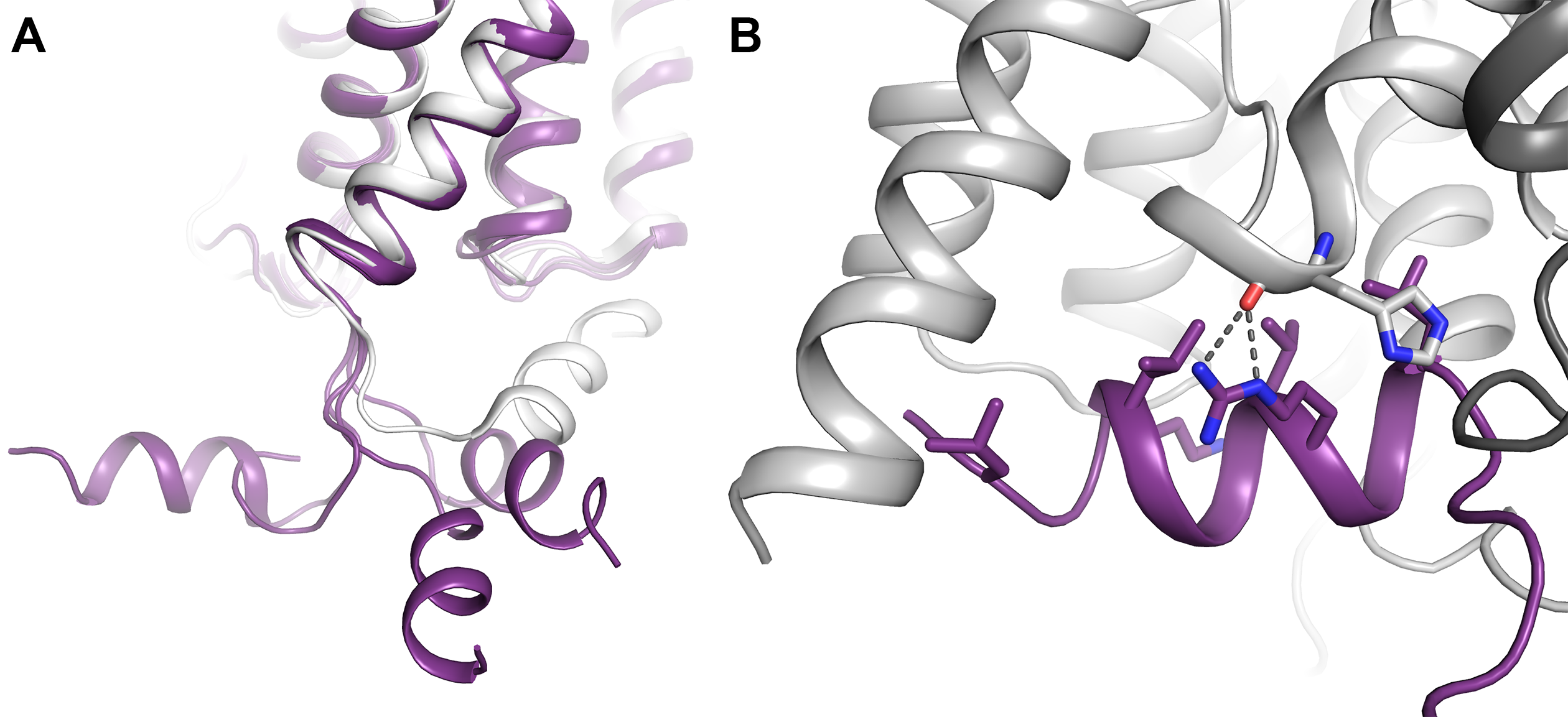

Supplement: Supplementary file 1 [file ijms-20-05351-s001.zip › Supplementary files/Figure S1.tif]

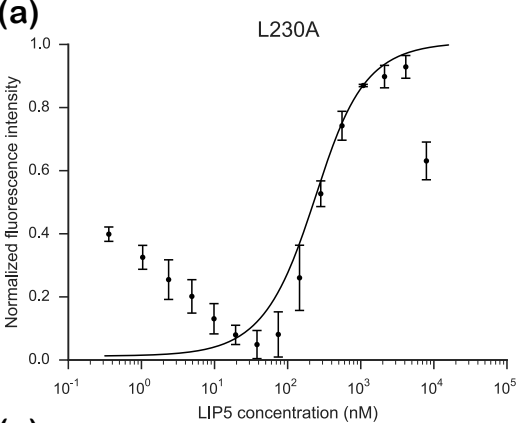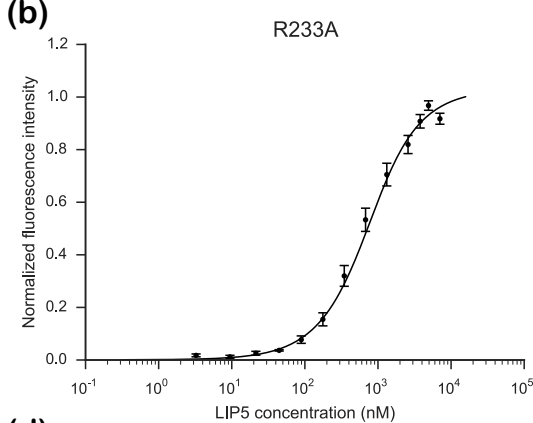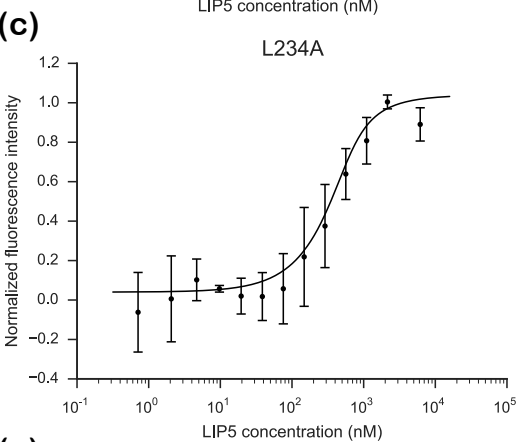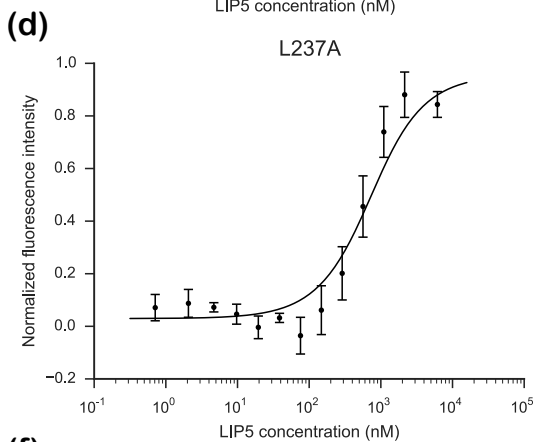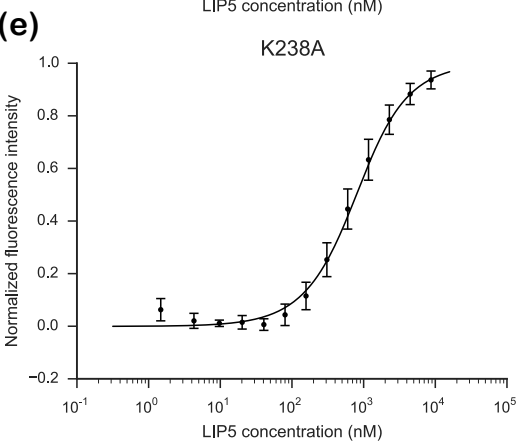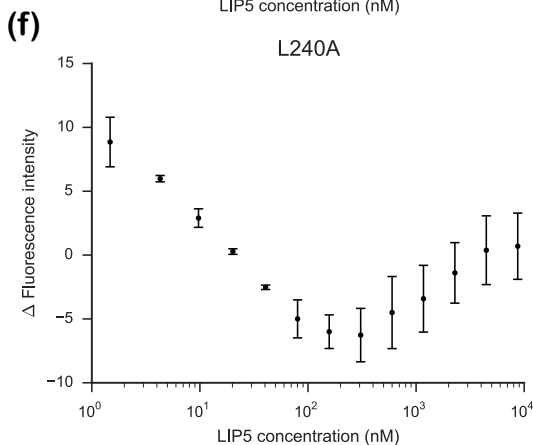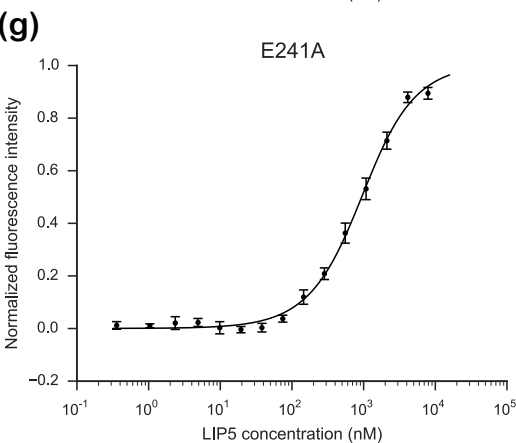

Supplement: Supplementary file 1 [file ijms-20-05351-s001.zip › Supplementary files/Figure S3.pdf]

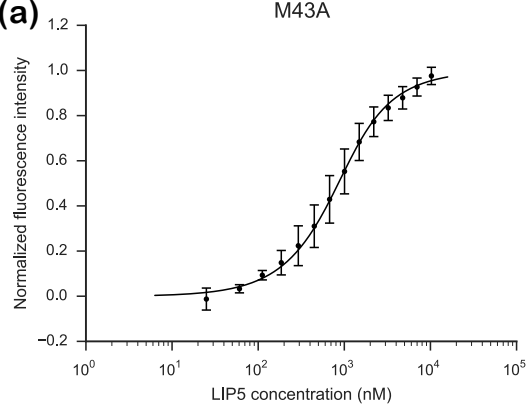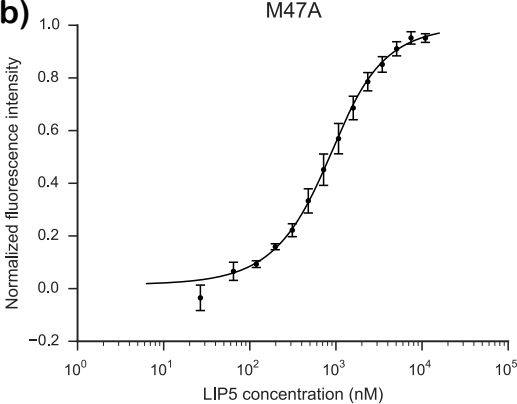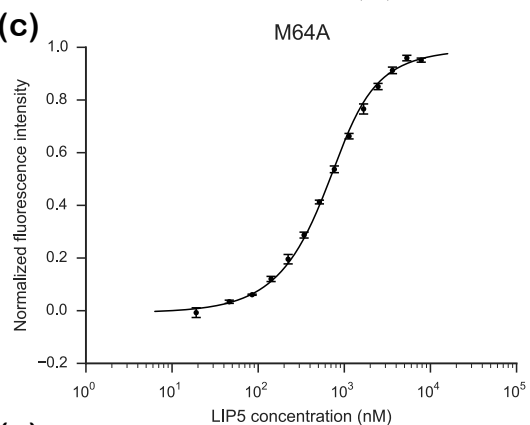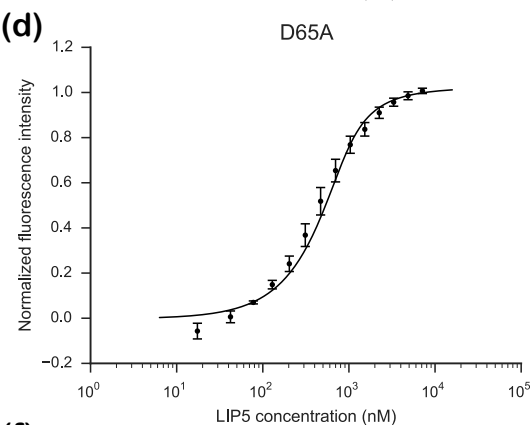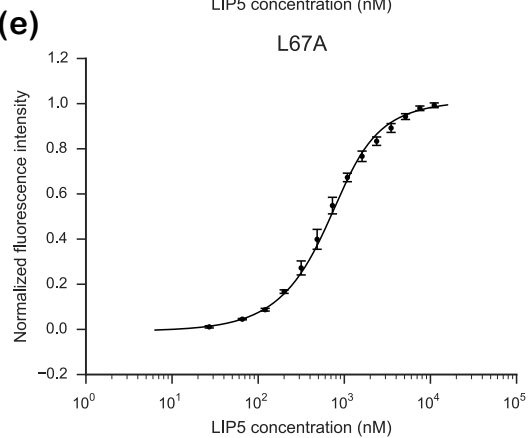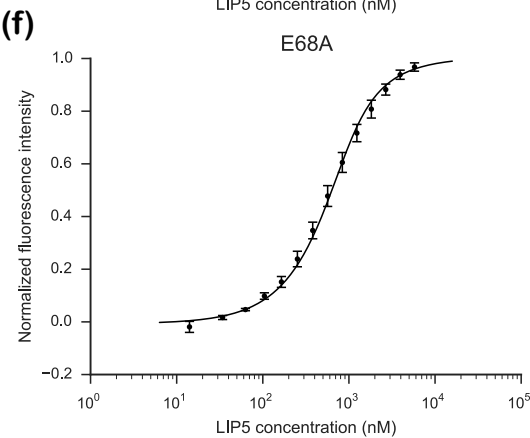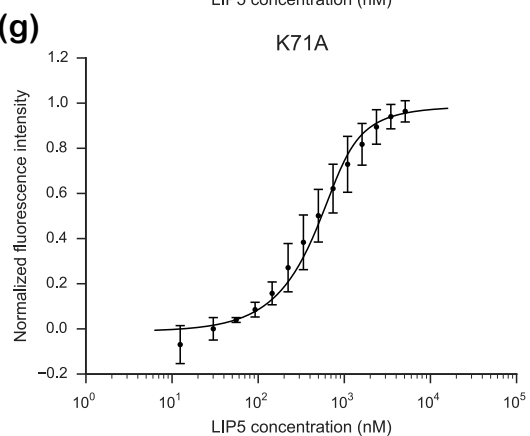

Supplement: Supplementary file 1 [file ijms-20-05351-s001.zip › Supplementary files/Figure S4.pdf]
